# Effectiveness of supplemental oxygenation to prevent surgical site infections: A systematic review with meta-analysis

**DOI:** 10.1590/1518-8345.6106.3648

**Published:** 2022-10-07

**Authors:** Eduardo Tavares Gomes, Fábio da Costa Carbogim, Rossana Sant’Anna Lins, Ruy Leite de Melo Lins-Filho, Vanessa de Brito Poveda, Vilanice Alves de Araujo Püschel

**Affiliations:** 1 Universidade de São Paulo, Escola de Enfermagem, São Paulo, SP, Brazil.; 2 Universidade Federal de Pernambuco, Hospital das Clínicas, Recife, PE, Brazil.; 3 Universidade Federal de Juiz de Fora, Departamento de Enfermagem, Juiz de Fora, MG, Brazil.

**Keywords:** Surgical Wound, Wound Infection, Patient Safety, Operative Surgical Procedures, Operating Room Nursing, Anesthesiology, Ferida Cirúrgica, Infecção da Ferida, Segurança do Paciente, Procedimentos Cirúrgicos Operatórios, Enfermagem de Centro Cirúrgico, Anestesiologia, Herida Quirúrgica, Infección de la Herida, Seguridad del Paciente, Procedimientos Quirúrgicos Operativos, Enfermería de Quirófano, Anestesiología

## Abstract

**Objective:**

to assess the effectiveness of supplemental oxygenation with high FiO_2_ when compared to conventional FiO_2_ in the prevention of surgical site infection.

**Method:**

an effectiveness systematic review with meta-analysis conducted in five international databases and portals. The research was guided by the following question: Which is the effectiveness of supplemental oxygenation with high FiO^2^ (greater than 80%) when compared to conventional FiO_2_ (from 30% to 35%) in the prevention of surgical site infections in adults?

**Results:**

fifteen randomized clinical trials were included. Although all the subgroups presented a general effect in favor of the intervention, colorectal surgeries had this relationship evidenced with statistical significance (I^2^=10%;*X^2^* =4.42; p=0.352).

**Conclusion:**

inspired oxygen fractions greater than 80% during the perioperative period in colorectal surgeries have proved to be effective to prevent surgical site infections, reducing their incidence by up to 27% (p=0.006). It is suggested to conduct new studies in groups of patients subjected to surgeries from other specialties, such as cardiac and vascular. PROSPERO registration No.: 178,453.

## Resumo

**Objetivo:**

avaliar a efetividade da oxigenação suplementar com FiO_2_ elevada comparada com FiO_2_ convencional na prevenção de infecção de sítio cirúrgico.

**Método:**

revisão sistemática de efetividade com metanálise em cinco bases de dados e portais internacionais. A pesquisa foi norteada pela pergunta: Qual a efetividade da oxigenação suplementar com FiO^2^ elevada (maior que 80%) comparada com FiO_2_ convencional (de 30 a 35%) na prevenção de infecção de sítio cirúrgico em adultos?

**Resultados:**

foram incluídos quinze ensaios clínicos randomizados. Embora todos os subgrupos tenham apresentado um efeito geral a favor da intervenção, as cirurgias colorretais tiveram essa relação evidenciada com significância estatística (I^2^=10%; *X^2^* =4,42; p=0,352).

**Conclusão:**

uma fração inspirada de oxigênio maior que 80% durante o período perioperatório em cirurgias colorretais tem se mostrado efetiva para prevenir a infecção de sítio cirúrgico, reduzindo sua incidência em até 27% (p=0,006). Sugere-se a realização de novos estudos em grupos de pacientes submetidos a cirurgias de outras especialidades, tais como cardíacas e vasculares. Registro PROSPERO: 178453.

## Resumen

**Objetivo:**

evaluar la efectividad de la oxigenación suplementaria con FiO_2_ elevada en comparación con la FiO_2_ convencional para prevenir la infección del sitio quirúrgico.

**Método:**

revisión sistemática de eficacia con metaanálisis en cinco bases de datos y portales internacionales. La investigación se guio por la pregunta: ¿Qué tan eficaz es la oxigenación suplementaria con FiO_2_ alta (más del 80%) en comparación con la FiO_2_ convencional (del 30 al 35%) para prevenir la infección del sitio quirúrgico en adultos?

**Resultados:**

se incluyeron quince ensayos clínicos aleatorizados. Aunque todos los subgrupos mostraron un efecto general a favor de la intervención, en las cirugías colorrectales esa relación tenía significancia estadística (I^2^=10%; *X*²=4,42; p=0,352).

**Conclusión:**

una fracción inspirada de oxígeno superior al 80% durante el perioperatorio en cirugías colorrectales ha demostrado ser eficaz en la prevención de la infección del sitio quirúrgico, reduciendo su incidencia hasta en un 27% (p=0,006). Se sugiere realizar más estudios en grupos de pacientes sometidos a cirugías en otras especialidades, como cardiaca y vascular. Registro PROSPERO: 178453.

**Highlights**

(1) The guidelines suggest perioperative hyperoxygenation for SSI prevention.

(2) Preliminary reviews did not find enough evidence for such recommendation.

(3) The previous reviews were published more than a decade ago.

(4) The current meta-analysis found evidence in favor of the intervention for colon surgeries.

## Introduction

Surgical Site Infection (SSI) refers to an infection that occurs after surgery at the incision or in the part of the body where the surgery was performed and can involve the skin, tissues, organs or material implanted within the first 30 days or within 90 days if prostheses are implanted^1^ ^-^ ^2^.

The Center for Disease Control (CDC), an American body for disease control, classifies SSIs as follows: superficial, when they involve the skin and subcutaneous tissue; deep, when they reach deeper incision tissues such as fascia and muscle; and organ/space, in cases involving deep regions beyond the fascia, which were exposed after the surgical procedure^1^ ^-^ ^2^.

SSI increases the risks for other complications, such as surgical wound dehiscence and sepsis, which can lead to second surgeries, increased hospitalization times and hospital costs, with the possibility of worsening the patients’ quality of life, which justifies making every possible effort to prevent this infection^3^ ^-^ ^7^.

In the latest updates of the main guidelines for the prevention of surgical site infection^1^ ^,^ ^8^ ^-^ ^10^, supplemental oxygenation has gained prominence, that is, maintenance of high inspired oxygen fractions (FiO_2_) in the perioperative period in order to prevent SSI.

The inspired oxygen fraction administered to the patient in the intraoperative and immediate postoperative periods is determined by the anesthesiologist, based on preoperative clinical criteria in the anesthetic technique used and on the patient’s response evaluated by monitoring the respiratory function. Hemoglobin oxygen saturation (SatO_2_) below 94% and associated with the patient’s previous clinical conditions are considered indications for increased FiO_2_. Even today, the possibility of offering greater oxygenation with the intention of preventing surgical site infection is not considered, and the indication is not widespread among anesthesiologists and nurses.

A previous search was carried out in the International Prospective Register of Systematic Reviews - PROSPERO, MEDLINE, Cochrane Database of Systematic Reviews and JBI Database of Systematic Reviews and Implementation Reports, and already existing systematic reviews related to the topic of interest were identified. The knowledge produced on the theme is systematized in reviews published since 2009^11^, which differ in terms of their results and in relation to the recommendation of perioperative hyperoxygenation for the prevention of SSI. The authors identified as a weakness the question of whether the analysis was performed based on only one type of surgery or on the fact that several types were grouped without performing any subgroup analysis^11^ ^-^ ^19^.

Thus, the current review advances knowledge by including in its analysis randomized clinical trials on the perioperative supplemental hyperoxygenation intervention, regardless of the surgical specialty, performing a meta-analysis by type of surgery, updating the diverse evidence on the theme and allowing for a critical reflection on the main guidelines for the prevention of SSIs. Furthermore, this study updates the knowledge by including important studies developed after the reviews found in the preliminary search, aiming to evaluate the effectiveness of supplemental oxygenation with high FiO_2_ when compared to conventional FiO_2_ in the prevention of surgical site infections.

## Method

A systematic review conducted according to the recommendations set forth by the Joanna Briggs Institute (JBI), and registered in the PROSPERO platform under No. 178,453. The search was conducted in October 2021. The research was guided by the PICO acronym: P-*Patients*: adult patients subjected to surgeries in general or from any specialty; I-*Intervention*: High inspired oxygen fraction (FiO_2_ greater than 80%) in the perioperative period; C-*Comparator*: Conventional inspired oxygen fraction (FiO_2_ 30%-35%) in the perioperative period; *O-Outcome*: Surgical Site Infection. It was based on this acronym that the following research question was prepared: Which is the effectiveness of supplemental oxygenation with high FiO_2_ (greater than 80%) when compared to conventional FiO_2_ (from 30% to 35%) in the prevention of surgical site infections in adults? The Preferred Reporting Items for Systematic Reviews and Meta-Analyses (PRISMA) were used to guide elaboration of this review^20^.

### Eligibility criteria

The studies included were those published from 2000 to September 2021, considering that an increase in the production on the topic is recorded from that period onwards. Studies with adult patients subjected to surgeries from any specialty were included. The intervention (FiO_2_ greater than 80%) and the comparator (FiO_2_ 30%-35%), with each description, considered that the inspired oxygen fraction had been maintained in this goal, regardless of the administration route and postoperative time. The outcome was occurrence of surgical site infection in up to ninety postoperative days. The eligibility criteria are similar to those of the main guidelines about the topic prior to this review.

### Information sources

The following databases were used to select the articles: National Library of Medicine (PubMed); Web of Science; Scopus Info Site (Scopus); *Literatura Latino-Americana e do Caribe em Ciências da Saúde*(LILACS) and Cumulative Index to Nursing and Allied Health Literature (CINHAL). Randomized clinical trials published in Portuguese, English or Spanish were included, which used supplemental oxygenation as a strategy to prevent surgical site infection. The non-inclusion criteria were as follows: studies with other surgical complications (such as granuloma, seromas or cellulitis), articles that dealt with oxygen therapy in other scenarios, editorials and letters to the editor.

### Search strategy

Descriptors from the Descriptors in Health Sciences (*Descritores em Ciências da Saúde*, DeCS) and from the Medical Subject Headings (MeSH) were selected for the search, namely: *Oxygen, Oxygenation*, and *Surgical Wound Infection*. Combined strategies were used in different ways with the purpose of achieving a broad search due to the characteristics of access to the databases selected, and having as guiding axes the study question and the inclusion criteria previously established according to the following combinations: Medline via PubMed: ((“*oxygenation*”) AND (“*surgical wound infection*”)); Scopus: *((“Surgical Wound Infection” ) AND (“Oxygenation”))*; Web of Science: (“*Surgical Wound Infection”) AND (“Oxygenation”);* LILACS: (mh*:(“infeccao da ferida cirurgica”))* OR (mh*:(“infeccao da ferida operatoria”))* AND (mh*:(“oxigenio”))*; CINAHL: MH *(surgical wound infection or surgical site infection)* AND MH *(oxygen therapy or oxygen treatment or oxygen* OR *oxygenation*). 

### Selection process

After the stage where the articles were identified in the databases, the titles and abstracts of each article were analyzed, as well as the keywords/descriptors. Subsequently, the references of all the articles were consulted to identify additional studies. 

The studies were selected by two reviewers with experience in review studies, independently and blindly, with consensus for inclusion of the articles. In turn, any and all disagreements were discussed with a third reviewer.

### Data extraction process

The first evaluation of the articles took place through their titles and abstracts and, subsequently, the full texts were read to extract the following data: title of the article, name of the journal, authors, country, language, year of publication, type of study, objective, study population, period of study, intervention, evaluation method, statistical analysis, result and conclusion. The web version of the *EndNote*™ software was used to organize the references found.

The studies selected were imported into the JBI System for the Unified Management, Assessment and Review of Information (JBI SUMARI; JBI, Adelaide, Australia) and evaluated in detail in relation to all the inclusion criteria as designed by the instrument for critical evaluation of studies. SUMARI is a software program developed to support systematic reviews and facilitate the entire review process, from development of the protocol to writing of the final report^21^. 

### Risk of bias assessment corresponding to the studies

The JBI Data Extraction Form for Experimental/Observational Studies instrument^22^ was used in the final critical evaluation of the articles. In this stage, both evaluators performed the methodological critical evaluation independently and the concepts attributed were considered when in agreement between both. Subsequently, the articles were included if they presented more than 70% agreement. Finally, the evaluators assessed the risks of bias.

### Effect measures

Synthesis of the results occurred in a narrative way and with a meta-analysis. The meta-analysis was prepared with the aid of the *SUMARI* online software program^21^. The results are summarized in the subgroup analysis (colorectal surgeries, C-sections and abdominal surgeries) by means of the Mantel-Haenszel model. Considering that the studies are homogeneous in terms of method, population by subgroup, intervention and outcome, the meta-analysis was prepared through the fixed-effects model^23^. The Relative Risk is presented with its Confidence Interval (CI) within the estimated limits equal to ±1.96 SE, where SE is the corresponding Standard Error value. The calculation of heterogeneity was performed by means of I^2^, considering that all studies have the same outcome. I^2^ values of 25%, 50% and 75% were used to define heterogeneity as low, moderate and high, respectively^24^.

## Results

**Figure 1:**
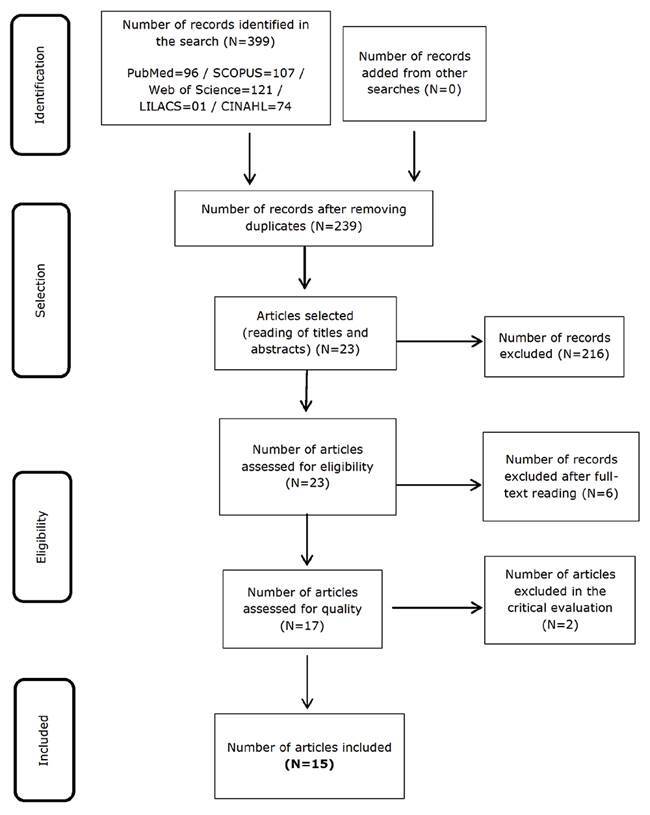
Flowchart corresponding to selection of the articles that comprised the analysis corpus according to PRISMA. São Paulo, Brazil, 2021

The search in the databases selected resulted in 399 articles, of which 160 were excluded for being duplicates. The number of articles excluded for not meeting the criteria after reading the titles and abstracts corresponded to n=216, namely: editorials, errata, responses, opinion, comments and letters to the editor (n=22); abstracts (n=5); literature reviews on the topic (n=14); study protocols (n=1); studies in the animal experimentation phase (n=2); publications in the veterinary field (n=2); articles that dealt with other interventions such as hyperbaric oxygenation, extracorporeal membrane oxygenation (ECMO), hypercarpnia, vacuum therapy and fluid administration, or antibiotic prophylaxis (n=170). One article was excluded because there was a retraction by the authors in the same journal acknowledging errors in the statistical analysis and methods that would preclude recognizing its findings^25^. The articles excluded after reading the full texts (n=16) for not answering the research question evaluated physiological, immunological and hemodynamic aspects, but did not directly or indirectly assess the *surgical site infection* outcome. Of these, one study was excluded for having being the only one that evaluated the intervention in trauma surgeries^26^, and another for focusing on the administration of nitrous oxide^27^. Seventeen articles were assessed by independent evaluators, with two exclusions. In the end, fifteen randomized clinical trials were included in the meta-analysis. Figure 1 describes the process corresponding to selection and inclusion of the articles.

Most of the articles included were from the United States (n=8; 57.15%), followed by Spain (n=2; 14.28%), Denmark (n=2; 14.28%), France (n=1; 7.14%) and India (n=1; 7.14%). The studies were conducted with patients subjected to colorectal surgeries (n=5; 35.71%), C-sections (n=4; 28.57%) and abdominal surgeries (n=6; 42.86%).

The results corresponding to the critical evaluation of the methodological quality of the randomized clinical trials are found in Figure 2. The studies reached scores from 78.6% to 100.0%, which can be considered low risks of bias.

**Figure 2:** Critical evaluation of the methodological quality of the randomized clinical trials, according to the JBI methodology. São Paulo, Brazil, 2021

Study	Q1^*^	Q2^†^	Q3^‡^	Q4^§^	Q5^||^	Q6^¶^	Q7^**^	Q8^††^	Q9^‡‡^	Q10^§§^	Q11^||||^	Q12^¶¶^	Q13^***^
Belda, et al., 2005^28^	U^†††^	Y^‡‡‡^	Y^‡‡‡^	Y^‡‡‡^	Y^‡‡‡^	Y^‡‡‡^	Y^‡‡‡^	Y^‡‡‡^	Y^‡‡‡^	Y^‡‡‡^	Y^‡‡‡^	Y^‡‡‡^	Y^‡‡‡^
Duggal, et al., 2013^29^	U^†††^	Y^‡‡‡^	Y^‡‡‡^	Y^‡‡‡^	Y^‡‡‡^	Y^‡‡‡^	Y^‡‡‡^	Y^‡‡‡^	Y^‡‡‡^	Y^‡‡‡^	Y^‡‡‡^	Y^‡‡‡^	Y^‡‡‡^
Ferrando, et al., 2020^30^	Y^‡‡‡^	Y^‡‡‡^	Y^‡‡‡^	Y^‡‡‡^	Y^‡‡‡^	Y^‡‡‡^	Y^‡‡‡^	Y^‡‡‡^	Y^‡‡‡^	Y^‡‡‡^	Y^‡‡‡^	Y^‡‡‡^	Y^‡‡‡^
Gardella, et al., 2008^31^	Y^‡‡‡^	Y^‡‡‡^	Y^‡‡‡^	Y^‡‡‡^	Y^‡‡‡^	Y^‡‡‡^	Y^‡‡‡^	Y^‡‡‡^	Y^‡‡‡^	Y^‡‡‡^	Y^‡‡‡^	Y^‡‡‡^	Y^‡‡‡^
Greif, et al., 2000^32^	Y^‡‡‡^	Y^‡‡‡^	Y^‡‡‡^	Y^‡‡‡^	Y^‡‡‡^	Y^‡‡‡^	Y^‡‡‡^	Y^‡‡‡^	Y^‡‡‡^	Y^‡‡‡^	Y^‡‡‡^	Y^‡‡‡^	Y^‡‡‡^
Kurz, et al, 2015^33^	Y^‡‡‡^	Y^‡‡‡^	Y^‡‡‡^	Y^‡‡‡^	Y^‡‡‡^	Y^‡‡‡^	Y^‡‡‡^	Y^‡‡‡^	Y^‡‡‡^	Y^‡‡‡^	Y^‡‡‡^	Y^‡‡‡^	Y^‡‡‡^
Mayank, et al., 2019^34^	Y^‡‡‡^	Y^‡‡‡^	Y^‡‡‡^	Y^‡‡‡^	Y^‡‡‡^	U^†††^	Y^‡‡‡^	Y^‡‡‡^	Y^‡‡‡^	Y^‡‡‡^	Y^‡‡‡^	Y^‡‡‡^	Y^‡‡‡^
Meyhoff, et al., 2009^35^	Y^‡‡‡^	Y^‡‡‡^	Y^‡‡‡^	Y^‡‡‡^	Y^‡‡‡^	Y^‡‡‡^	Y^‡‡‡^	Y^‡‡‡^	Y^‡‡‡^	Y^‡‡‡^	Y^‡‡‡^	Y^‡‡‡^	Y^‡‡‡^
Staehr, et al., 2011^36^	Y^‡‡‡^	Y^‡‡‡^	Y^‡‡‡^	Y^‡‡‡^	Y^‡‡‡^	Y^‡‡‡^	Y^‡‡‡^	Y^‡‡‡^	Y^‡‡‡^	Y^‡‡‡^	Y^‡‡‡^	Y^‡‡‡^	Y^‡‡‡^
Thibon, et al., 2012^37^	U^†††^	U^†††^	Y^‡‡‡^	Y^‡‡‡^	Y^‡‡‡^	U^†††^	Y^‡‡‡^	Y^‡‡‡^	Y^‡‡‡^	Y^‡‡‡^	Y^‡‡‡^	Y^‡‡‡^	Y^‡‡‡^
Wadhwa, et al., 2014^38^	Y^‡‡‡^	Y^‡‡‡^	Y^‡‡‡^	Y^‡‡‡^	Y^‡‡‡^	Y^‡‡‡^	Y^‡‡‡^	Y^‡‡‡^	Y^‡‡‡^	Y^‡‡‡^	Y^‡‡‡^	Y^‡‡‡^	Y^‡‡‡^
Williams, et al., 2013^39^	Y^‡‡‡^	Y^‡‡‡^	Y^‡‡‡^	Y^‡‡‡^	Y^‡‡‡^	Y^‡‡‡^	Y^‡‡‡^	Y^‡‡‡^	Y^‡‡‡^	Y^‡‡‡^	Y^‡‡‡^	Y^‡‡‡^	Y^‡‡‡^
Scifres, et al., 2011^40^	Y^‡‡‡^	Y^‡‡‡^	Y^‡‡‡^	N^§§§^	N^§§§^	N^§§§^	Y^‡‡‡^	Y^‡‡‡^	Y^‡‡‡^	Y^‡‡‡^	Y^‡‡‡^	Y^‡‡‡^	U^†††^
Mayzler, et al., 2005^41^	Y^‡‡‡^	U^†††^	Y^‡‡‡^	U^†††^	U^†††^	Y^‡‡‡^	Y^‡‡‡^	Y^‡‡‡^	Y^‡‡‡^	Y^‡‡‡^	Y^‡‡‡^	Y^‡‡‡^	Y^‡‡‡^
Pryor, et al., 2004^42^	Y^‡‡‡^	Y^‡‡‡^	Y^‡‡‡^	Y^‡‡‡^	Y^‡‡‡^	Y^‡‡‡^	Y^‡‡‡^	Y^‡‡‡^	Y^‡‡‡^	Y^‡‡‡^	Y^‡‡‡^	Y^‡‡‡^	Y^‡‡‡^
%	78.6	85.7	100.0	85.7	85.7	78.6	100.0	100.0	100.0	100.0	100.0	100.0	92.8

Questions from the JBI critical appraisal instrument: ^*^Q1 = 1. Was true randomization used for allocation of the participants to the treatment groups?; ^†^Q2 = Was allocation to the treatment groups concealed?; ^‡^Q3 = Were the treatment groups similar at the baseline?; ^§^Q4 = Were the participants blinded to treatment allocation?; ^||^Q5 = Were those delivering treatment blinded to treatment allocation?; ^¶^Q6 = Were the outcome evaluators blinded to treatment allocation?; ^**^Q7 = Were the treatment groups treated identically to the intervention of interest?; ^††^Q8 = Was follow-up complete and, if not, were the differences between the groups in terms of follow-up adequately described and analyzed?; ^‡‡^Q9 = Was follow-up complete and, if not, were differences between the groups in terms of follow-up adequately described and analyzed?; ^§§^Q10 = Were the results measured in the same way for the treatment groups?; ^||||^Q11 = Were the results measured reliably?; ^¶¶^Q12 = Was an appropriate statistical analysis used?; ^***^Q13 = Was the study design appropriate and were any deviations from the standard RCT design (individual randomization, parallel groups) considered in conducting and analyzing the study?; ^†††^U: Uncertain; ^‡‡‡^Y: Yes; ^§§§^N: No

The Figure 3 presents a summary of the studies included. In relation to the surgery, the surgical site infection rate was calculated in the following ranges: colorectal surgeries, from 5.2% to 55.3%; abdominal surgeries, from 6.6% to 31.0%; and C-sections, from 5.3% to 14.5% (Figure 3).

**Figure 3:** Characteristics of the randomized clinical trials (level of evidence 1c) about perioperative supplemental oxygenation included in the review. São Paulo, Brazil, 2021

Author/Journal/Year	Types of Surgery	Intervention - FiO_2_^*^ 80% (n)	Control - FiO_2_^*^ 30% (n)	SSI^†^ rates
Ferrando, et al. Br J Anaesth. 2020.^30^	Abdominal	IO^‡^ (OTI^§^) and PO^||^ (rebreathing face mask for 3 h) n=371	IO^‡^ (OTI^§^) and PO^||^ (Venturi mask for 3 h) n=369	IG^¶^=8.9% CG^**^=9.4% (p=0.9)
Mayank, et al. J Gastrointest Surg. 2019.^34^	Colorectal	IO^‡^ (OTI^§^) and PO^||^ (rebreathing face mask for 6 h) n=47	IO^‡^ (OTI^§^) and PO^||^ (Venturi mask for 6 h) n=47	IG^¶^=55.3% CG^**^=0.4% (p=0.215)
Kurz, et al. Br J Anaesth. 2015.^33^	Colorectal	IO^‡^ (OTI^§^) and PO^||^ (rebreathing face mask for 1 h) n=285	IO^‡^ (OTI^§^) and PO^||^ (Venturi mask for 1 h) n=270	IG^¶^=15.6% CG^**^=15.8% (p=0.201)^††^
Wadhwa, et al. Anest Analg. 2014.^38^	Bariatric	IO^‡^ (OTI^§^) and PO^||^ (10 L/min by rebreathing face mask or 15 L/min via nasal cannula, for 12 h to 16 h) n=202	IO^‡^ (OTI^§^) and PO^||^ (nasal cannula 2 L/min) n=198	IG^¶^=7.9% CG^**^=9.1% (p=0.80)^††^
Thibon, et al. Anesthesiology. 2012.^37^	Abdominal, gynecological and breast	IO^‡^ (OTI^§^) and PO^||^ (nebulization mask, it does not describe the postoperative time, it suggests that it was only during the stay in the post-anesthetic recovery room) n=226	n=208	IG^¶^=6.6% CG^**^=7.2% (p=0.81)
Staehr, et al. Anesthesiol. 2011.^36^	Abdominal, in obese individuals	IO^‡^ (OTI^§^) and PO^||^ (nebulization mask with variable supply, not reported, for 2 h) n=102	n=111	IG^¶^=26% CG^**^=31% (p=0.4)
Scifres, et al. Am J Obstet Gynecol. 2011.^43^	C-section	n=288 Intraoperative and postoperative intervention (nasal cannula 2 L/min X 10 L/min by face mask, for 2 h)	n=297	IG^¶^=12.2% CG^**^=8.8% (p=0.28)
Meyhoff, et al. JAMA. 2009.^35^	Abdominal	IO^‡^ (OTI^§^) and PO^||^ (nebulization mask with variable supply, not reported, for 2 h) n=685	n=701	IG^¶^=19.1% GC^**^=20.1% (p=0.81)
Belda, et al. JAMA. 2005.^28^	Colorectal	IO^‡^ (OTI^§^) and PO^||^ (nebulization mask with variable supply, not reported, for 6 h) n=148	n=143	IG^¶^=14.9% CG^**^=24.4% (p=0.13)
Mayzler, et al. Minerva Anestesiol. 2005.^41^	Colorectal	IO^‡^ (OTI^§^) and PO^||^ (non-rebreathing face mask, for 2 h) n=19	n=19	IG^¶^=12.5% CG^**^=15.7% (p=0.53)
Pryor, et al. JAMA. 2004.^42^	Abdominal	IO^‡^ (OTI^§^) and PO^||^ (non-rebreathing face mask 10 L/min for 2 h) n=80	IO^†^ (OTI^‡^) and PO^||^ (nasal cannula 4 L/min for 2 h) n=80	IG^¶^=11.3% CG^**^=25% (p=0.13)
Greif, et al. N Engl J Med. 2000.^32^	Colorectal	IO^‡^ (OTI^§^) and PO^||^ (non-rebreathing face mask, for 2 h) n=250	n=250	IG^¶^=5.2% CG^**^=11.2% (p=0.01)
**Regional Anesthesia**				
Duggal, et al. Obstet Gynecol. 2013.^29^	C-section	IO^‡^ and PO^||^ (nebulization mask 10 L/min, for 1 h) n=415	n=416	IG^¶^=5.5% CG^**^=5.8% (p=0.98)
Williams, et al. Am J Perinatol. 2013.^39^	C-section	IO^‡^ and PO^||^ (nebulization mask with variable offer, not reported, for 2 h) n=77	n=83	IG^¶^=13% CG^**^=14.5% (p=0.82)^††^
Gardella, et al. Obstet Gynecol. 2008.^31^	C-section	IO^‡^ and PO^||^ (non-rebreathing mask, for 2 h) n=69	n=83	IG^¶^=14% CG^**^=25% (p=0.13)^††^

*FiO_2_ = Inspired Oxygen Fraction; ^†^SSI = Surgical Site Infection; ^‡^IO = Intraoperative; ^§^OTI = Orotracheal Intubation; ^||^PO = Postoperative; ^¶^IG = Intervention Group; ^**^CG = Control Group; ^††^Terminated before time (futility criterion)

**Figure 4:**
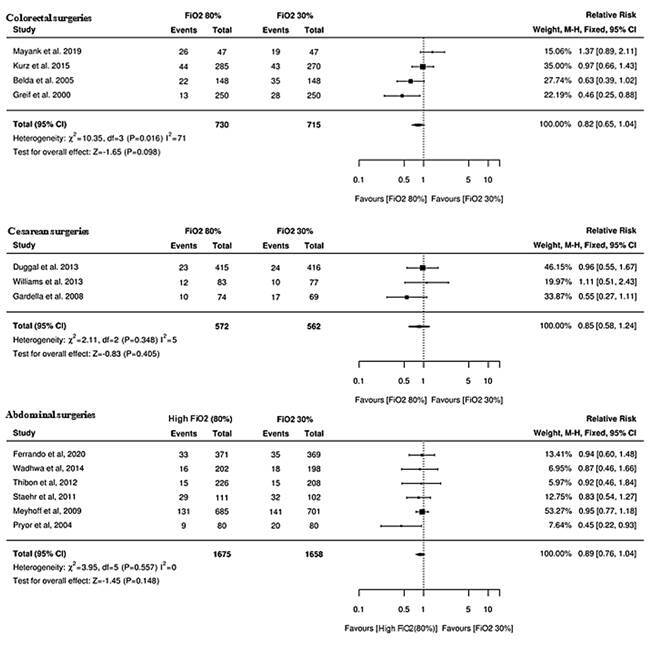
Forest plot showing incidence and relative risk of surgical site infections by subgroup (colorectal, cesarean and abdominal surgeries) when compared to high inspired oxygen fraction (FiO_2_>=80%) versus traditional supply (FiO_2_: 30%-35%). São Paulo, Brazil, 2021.

The meta-analysis was performed by means of subgroups, namely: colorectal surgeries, 5 RCTs (N=1,483 participants); C-sections, 4 RCTs (n=1,719 participants) and abdominal surgeries, 6 RCTs (N=3,333 participants). The subgroup analysis was necessary to ensure clinical homogeneity. Heterogeneity was considered low for the subgroup of abdominal surgeries (I^2^=0.0%; *X*²=3.97; p=0.557), for the C-sections (I^2^=35%; *X*²=4.61; p=0.202) and for the colorectal surgeries (I^2^=10%; *X^2^* =4.42; p=0.352), with only the last estimate being considered statistically significant (Figure 4). Although all the subgroups presented a general effect in favor of the intervention, colorectal surgeries had this relationship evidenced with statistical significance (RR=0.73; 95% CI=0.58-0.91; p=0.006).

## Discussion

To prevent surgical site infections, it is essential to optimize the perioperative conditions, as the first hours after exposure of the surgical site to bacterial contamination are fundamental to avoid infection^43^. The findings on perioperative oxygen supplementation have a potential to contribute by bringing diverse evidence to the adoption of this practice in the prevention of surgical site infection.

The partial oxygen pressure is usually low in wounds and anastomoses at the end of a surgery, decreasing the body’s defenses against bacteria, reducing the activity of neutrophils and disfavoring tissue healing^14^ ^,^ ^22^ ^-^ ^27^ ^,^ ^43^ ^-^ ^44^. Tissue hypoxia reduced production of collagen and revascularization, which are necessary for tissue repair^18^ ^,^ ^32^ ^,^ ^43^ ^-^ ^45^. Perioperative and wound arterial oxygen pressure (PaO_2_) can be increased by a higher inspiratory oxygen fraction^14^ ^,^ ^32^ ^,^ ^45^, and hyperoxygenation may also be related to the optimization of the effect of some antibiotics^32^. However, the inspired fraction is not always related to better oxygenation in the surgical wound, due to dependence on other clinical factors of the patient and related to anesthesia. 

The benefit of tissue oxygenation has been studied through clinical trials that evaluated the infection outcome^28^ ^-^ ^35^ ^,^ ^37^ ^-^ ^38^ ^,^ ^40^ ^-^ ^42^ ^,^ ^45^ ^-^ ^50^, being incorporated into the guidelines for the practices to prevent Surgical Site Infection (SSI) based on the most current versions and since 2016^1^ ^,^ ^8^ ^-^ ^10^.

Although national studies have not yet evaluated high FiO_2_ as a risk factor for SSI^3^ ^-^ ^4^ ^,^ ^42^, a research study included the supplemental oxygenation strategy in a care bundle for obese patients subjected to bariatric surgery, which was related to the lower incidence of SSI^48^. However, this study did not describe how the intervention was performed in the postoperative period or how long it was maintained^48^. 

Regarding the methodological issue, it was observed that for this type of study it was possible, in all cases, to blind of the patient and the SSI evaluator in the postoperative period; however, it was not possible to blind the anesthesiologist, as mentioned by some authors^28^ ^-^ ^29^ ^,^ ^31^ ^,^ ^33^ ^,^ ^37^ ^,^ ^42^ ^,^ ^45^ ^,^ ^50^. In addition to that, it was evident that the multicenter studies presented larger samples, which significantly impacted on heterogeneity and on the results of the meta-analysis^33^ ^,^ ^35^ ^,^ ^37^ ^-^ ^38^ ^,^ ^41^ ^-^ ^42^. It is also noted that, after the evaluation of a partial, initial sample, four studies were terminated by the futility criterion because it was considered by statistical analysis that they would not find different results if they were continued^29^ ^,^ ^33^ ^,^ ^41^ ^-^ ^42^ ^,^ ^50^. 

In the meta-analysis there was low heterogeneity for the subgroup of the colorectal surgeries (I^2^=10%; *X^2^* =4.42; p=0.352). Although not high, clinical heterogeneity is due to the intervention, as the studies maintained a variable time of supplemental oxygenation in the postoperative period. In addition to that, from a methodological point of view, the discrepancy in the sample size of some studies in each subgroup with weights much higher than the others, or the period for evaluation of the surgical wound, can also interfere with heterogeneity.

The studies analyzed maintained the routine of only including patients who underwent adequate antibiotic prophylaxis, reducing this sample selection bias^12^. In addition to that, most of the study protocols provided for blinding of the patient and of the evaluator of the wounds in the postoperative period, although not blinding the anesthesiologist, in order to ensure maintenance of oxygen supply according to the randomized group^28^ ^-^ ^29^ ^,^ ^31^ ^,^ ^33^ ^,^ ^37^ ^,^ ^41^ ^-^ ^42^ ^,^ ^45^. 

The studies with C-sections presented a limitation due to the use of epidural or rachidian anesthesia. These surgeries are generally performed with the use of masks or nasal catheters, which hinder maintenance of a standard and constant FiO_2_, as is the case in general anesthesia with orotracheal intubation^16^ ^,^ ^45^ ^,^ ^50^. Oxygenation with a mask or nasal catheter is a limiting factor for these studies due to the variability of mask models and fits, differences in tidal volume *per* patient, failures in equipment and accessories, conversion to general anesthesia (not considered in the studies) and, perhaps the most important, deficient fit of the mask to the face with significant oxygen leakage^16^ ^,^ ^45^ ^,^ ^50^. In the studies referring to abdominal and colorectal surgeries, the patients undergo general anesthesia and maintain greater sedation in the immediate postoperative period, ensuring better adherence to the use of face masks. 

In addition to the type of anesthesia associated, the effect of hyperoxygenation may have been better observed in colorectal surgeries because they are contaminated, when compared to cesarean surgeries. The surgeries presented surgical site infection rates proportional to the contamination degree^3^ ^-^ ^4^. 

The assessment of hyperoxygenation was concentrated on two large groups of procedures, namely: gastrointestinal tract surgeries and gynecological surgeries. A publication that did not comprise the sample due to its methodological design (a series of ten cases) evaluated vascular surgeries and observed that high FiO_2_ maintained greater tissue oxygenation after arterial clamping^48^. 

Perioperative hyperoxia promotes cellular hyperoxia, shifting the balance of the intracellular reactions for excessive production of reactive oxygen species, such as in relation to hydrogen peroxide and superoxide anions and, consequently, increasing oxidative stress^51^. Oxidative stress promotes cell injury and death with potential pulmonary and neuronal toxicity and increase in the risk of kidney and heart failure^50^. Consequently, the studies that assess this intervention should consider the risks of complications in their outcomes Of the studies included, few reported having investigated these secondary outcomes and that did not occur significantly, although other studies are already evidencing that the risk of lung injury, as is the case in atelectasis, renal and theoretical myocardial has no evidence in the clinic^51^ ^-^ ^56^. Probably, the time of perioperative hyperoxygenation is not enough to have lung injury, when compared to critically-ill patients on mechanical ventilation, as well as the time of orotracheal intubation is shorter, minimizing the incidence of atelectasis^50^. 

The studies included in the review proved to be inconclusive to guide a change in the practice. The recommendations of the main Surgical Site Infection prevention guidelines on this topic report that, for patients with normal pulmonary function subjected to general anesthesia with endotracheal intubation, an increased inspired oxygen fraction (FiO_2_) should be administered intraoperatively and post-extubation in the immediate postoperative period^1^ ^,^ ^8^ ^-^ ^10^. Only one guideline indicates a time of supplemental oxygen postoperative administration from 2 to 6 hours^10^ and none of them guides the form of administration, as they only leave the inspired oxygen fraction greater than 80% as a goal^1^ ^,^ ^8^ ^-^ ^10^. Two guidelines reinforce that, in order to optimize oxygen delivery to the tissues, perioperative normothermia and adequate volume replacement must be maintained^1^ ^,^ ^9^. Only the *APIC* guideline emphasizes that the data are stronger for the colorectal surgeries, as can be found both in this meta-analysis and in others^11^ ^,^ ^13^ ^,^ ^15^ ^,^ ^19^. 

To change the practice, due to the potential risks that have not yet been well clarified in the studies considered in this review and in the current *guidelines* for SSI prevention, it should first be considered that normovolemia, normotension, normothermia, normoglycemia and normoventilation can be effective in the prevention of SSI and safely applied in these cases^56^. 

Finally, it can be asserted that the perspective about the current SSI prevention guidelines has been expanded after this discussion. The limitation of this review is the fact that segmentation into subgroups, although necessary to increase validity of the findings, reduces heterogeneity and the total sample size.

## Conclusion

Providing inspired oxygen fractions greater than 80% during the perioperative period in colorectal surgeries can be effective to prevent SSI, reducing its incidence by up to 27% (p=0.006). It is suggested to conduct new studies in groups of patients subjected to surgeries from other specialties, such as cardiac and vascular.
